# Common Variation Neighbouring Micro-RNA 22 Is Associated with Increased Left Ventricular Mass

**DOI:** 10.1371/journal.pone.0055061

**Published:** 2013-01-25

**Authors:** Andrew R. Harper, Bongani M. Mayosi, Antony Rodriguez, Thahira Rahman, Darroch Hall, Chrysovalanto Mamasoula, Peter J. Avery, Bernard D. Keavney

**Affiliations:** 1 Faculty of Medical Sciences, Newcastle University, Newcastle upon Tyne, United Kingdom; 2 Institute of Genetic Medicine, Newcastle University, Newcastle upon Tyne, United Kingdom; 3 Department of Medicine, Groote Schuur Hospital and University of Cape Town, Cape Town, South Africa; 4 Department of Molecular & Human Genetics, Baylor College of Medicine, Houston, Texas, United States of America; 5 School of Mathematics & Statistics, Newcastle University, Newcastle upon Tyne, United Kingdom; South Texas Veterans Health Care System and University Health Science Center San Antonio, United States of America

## Abstract

**Aims:**

Previous genome-wide linkage analysis has suggested that chromosomal region 17p13.3 may harbour genes influencing left ventricular mass (LVM) in man. To date, the genetic factors accounting for LVM variability remain largely unknown but a non-coding RNA gene within this region, micro-RNA 22 *(miR-22)*, has been implicated in cardiac hypertrophy and heart failure in animal models. We thus investigated the relationship between common genetic polymorphisms surrounding *miR-22* and left ventricular mass in a family-based association study.

**Methods and Results:**

We studied a cohort of 255 families comprising 1,425 individuals ascertained via a hypertensive proband. Ten single nucleotide polymorphisms which together tagged common genetic variation surrounding the *miR-22* gene were genotyped. There was evidence of association between the rs7223247 polymorphism, which lies within the 3′UTR of a gene of unknown function, TLCD2, immediately downstream from miR-22, and left ventricular mass determined by Sokolow-Lyon voltage (Bonferroni corrected *p-*value = 0.038). The T allele at rs7223247 was associated with an 0.272 standard deviation higher Sokolow-Lyon voltage. Genotype was responsible for ∼1% of the population variability in LVM.

**Conclusions:**

Genotype at the rs7223247 polymorphism affects left ventricular mass determined by Sokolow-Lyon voltage. The neighbouring genes *miR-22* and *TLCD2* are strong candidates to account for this observation.

## Introduction

Left ventricular hypertrophy is a strong independent predictor of cardiovascular morbidity and mortality. Left ventricular mass (LVM) measured using either the electrocardiogram (ECG) or by echocardiography has substantial heritability [1], but thus far the genes responsible involved remain largely unidentified. LVM measured using a heritable electrocardiographic derivative, Sokolow-Lyon voltage (SLV; obtained by summation of the S-wave voltage in ECG lead V_1_ and the R-wave voltage in lead V_5_) is an independent predictor of mortality [2]. Previous evidence from a family-based study suggested genetic linkage of ECG-voltage derived LVM to chromosomal region 17p13.3 (LOD score = 2.67; p = 0.0002) [3].

MicroRNAs (miRNAs) are a group of small non-coding RNA molecules involved in posttranscriptional gene regulation. During the last ten years many miRNAs have been identified as major regulators of cardiac hypertrophy [4], [5]. Within rat cardiomyocytes, phenotypic screening identified microRNA 22 *(miR-22)*, which is located within the region of 17p13 showing linkage to LVM in man, as a pro-hypertrophic modulating miRNA [6]–[8]. Gain of function mouse models support a role for *miR-22* as a mediator of LV hypertrophy traits in cardiac myocytes [8]. Interestingly, the recently implicated LVM human gene, osteoglycin was identified as a downstream target of *miR-22* mediating cardiac fibroblast activation [9], [10]. Moreover, targeted deletion of *miR-22* sensitizes mice to cardiac decompensation and LV dilatation when subjected to pressure overload [8]. *miR-22* is therefore a strong candidate gene influencing LVM variation in man.

**Figure 1:**
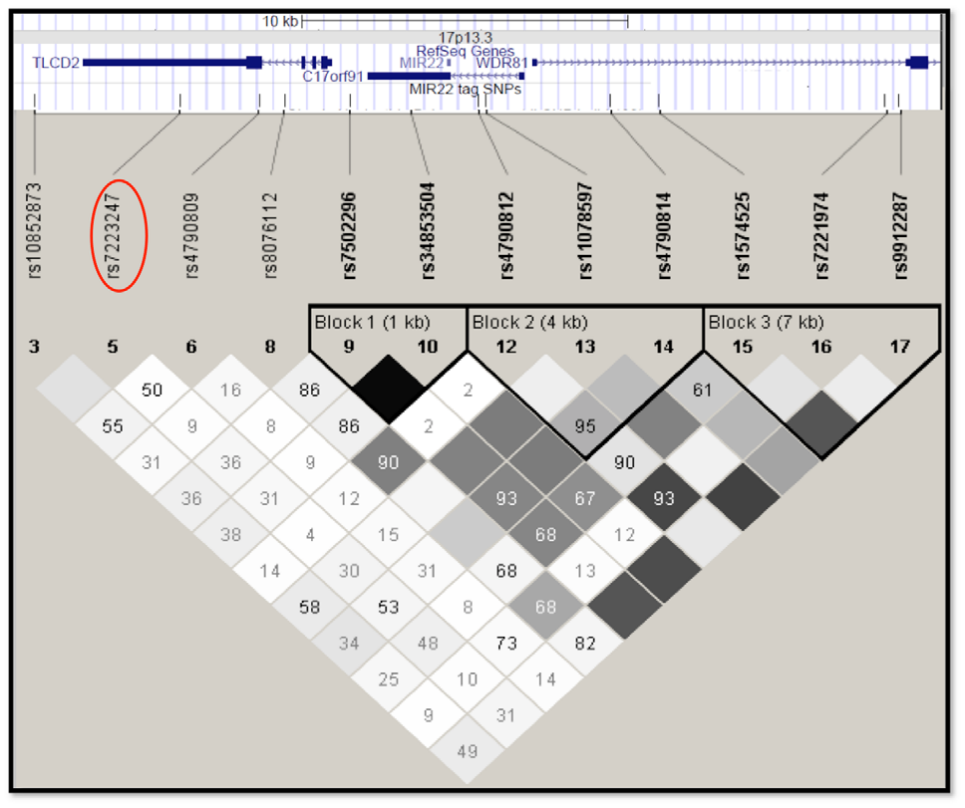
SNPs at the *miR-22* locus. All SNP locations are indicated by lines on the *miR-22* schematic (17p13.3/30.0 kb) derived from the UCSC genome browser (http://www.genome.ucsc.edu/). The Haploview output is directly related to the schematic, with the linkage disequilibrium relationships between HapMap Phase 3 SNPs in the region shown, indicating the three principal haplotype blocks. Darker squares represent higher R-squared between markers. rs7223247 is circled in red. SNPs chosen for genotyping (at threshold MAF = 0.05, r^2^ = 0.8) are enclosed within green boxes.

*miR-22* lies within the 5′ untranslated region of the open reading frame C17orf91, and is flanked by the expressed genes Tram-Lag-CLN8 domain 2 *(TLCD2)* and WD repeat 81 *(WDR81)* at chromosome 17p13.3 (Figure 1). The *TLCD2* gene codes for a 264 amino acid long helical transmembrane protein, which belongs to a large family of genes containing a Tram-Lag-CLN8 domain. The function of *TLCD2* is unknown, however it has been postulated that it may have a role in both lipid metabolism and ceramide synthesis [11]. Genes associated with these pathways have previously been implicated in the development of cardiac hypertrophy and its consequences [12]; for example ceramide accumulation contributes to heart failure in patients with cardiac hypertrophy [13]–[15]. Thus, *TLCD2* is also a candidate gene possibly influencing LVM in the linked region of 17p13.3. *WDR81* encodes a multi-domain transmembrane protein predominantly expressed in the brain. Mutations in the gene are associated with autosomal recessive cerebellar ataxia and other neurological conditions. Since these conditions are not associated with left ventricular hypertrophy, *WDR81* is a less strong candidate in the region than *TCLD2* and *miR-22*.

We conducted an association study of SNPs tagging common genetic variation in *miR-22* and *TCLD2* in a cohort of families ascertained via a proband with hypertension and phenotyped for LVM. This cohort had previously shown evidence of genetic linkage of LVM to the *miR-22/TCLD2* region; candidate-gene based fine-mapping within linked regions is an accepted strategy to discover genetic associations with complex diseases [16].

## Methods

### Ethics Statement

Written informed consent was obtained from all participants prior to enrolment. The study was approved by the Central Oxford Research Ethics Committee and Newcastle and North Tyneside Local Research Ethics Committee. The study was conducted according to the principles of the Declaration of Helsinki.

### Cohort Ascertainment and Phenotyping

Between 1993–1996 two hundred and forty-eight British Caucasian families were collected for a quantitative genetic investigation of LVM and other cardiovascular risk factors as previously described [17]. In summary, families were identified through essential hypertensive probands, categorised within the upper 5% of the population. Patients with secondary hypertension were excluded. Stringent inclusion criteria were employed. Suitable patients required daytime ambulatory blood pressure readings exceeding 140 mmHg systolic and 90 mmHg diastolic; or three clinic blood pressure measurements greater than 160/95 mmHg; or receiving treatment with a minimum of two antihypertensive drugs. Enrolment within the study cohort was conditional on appropriate family structure. Where one parent of the sibship was available to give blood for DNA analysis, families required at least three siblings clinically assessable for blood pressure measurement. Where no parent was available for DNA analysis, families required at least four assessable siblings. Quantitatively assessed sibships were recruited either in the generation of the proband or his/her offspring. If individuals from the sibship were classified as being hypertensive, spouses and offspring of the hypetensive sibs were also collected. Employing this recruitment strategy resulted in mainly nuclear families and some extended families, as previously described [18].

Participants underwent 24 hour ambulatory blood pressure monitoring, using the A&D TM2421 monitor according to a previously described protocol [19]. Detailed medical and lifestyle histories were recorded alongside anthropometric measurements of height, weight, waist and hip circumferences, using standard methods [20]. DNA was extracted from blood samples using standard methods. Families were recalled for additional cardiovascular phenotyping in 1999–2001, where resting 12-lead electrocardiographic measurements were recorded and SLV (S wave in V_1_ + R wave in V_5_) calculations performed according to standard protocols previously described [21]. As previously reported, LVM measured by electrocardiography in the cohort was higher than previously reported in the unselected general population, indiciating that our proxy selection for increased LVM through familial hypertension was successful [22].

### Genotyping

Tag SNPs within the *miR-22/TCLD2* region (±15 kb to incorporate flanking transcriptional regulating sites) were identified with reference to phase 3 HapMap CEU data (http://www.hapmap.org). Three major haplotype blocks existed within the region. The Tagger utility, within Haploview version 4.2, identified ten tag SNPs (Inclusion criteria: Hardy-Weinberg p-value cutoff = 0.001, minimum genotype = 75%, Mendelian errors = 0.05, minimum minor allele frequency = 0.05, r^2^ = 0.8). The LD pattern and location of SNPs in the region is shown in Figure 1. Multiplex genotyping was completed for 8 SNPs using Sequenom iPLEX. Validated Applied Biosciences™ TaqMan® SNP genotyping assays were implemented for 2 remaining SNPs using the Applied Bioscience™ 7900 HT fast RT-PCR system. Allelic discrimination algorithms were implemented to analyse data using Applied Bioscience™ SDS v2.3 software; with automatic genotype calls manually altered appropriately. Genotyping was carried out blinded to phenotypic information.

### Statistical Analysis

PEDSTATS was employed to determine Mendelian inheritance of all the genotypes, and Hardy-Weinberg equilibrium for each marker [23]. Phenotypes were assessed for Normality. All variables required log-transformation to adequately conform to a Normal distribution (P>0.05). Phenotypes were adjusted for the significant covariates, including age, systolic BP, weight, waist-hip ratio and height using multiple step-wise linear regression, as previously described [24]. Normalised residual values underwent quantitative trait genetic association analysis, implemented using MERLIN version 1.1.2 [25]. This approach allows consideration of shared polygenic effects shared between family members. FASTSNP (http://fastsnp.ibms.sinica.edu.tw) was utilised to determine the functional relevance of identified SNPs. We corrected our p-values for multiple testing using a Bonferroni approach; although this is likely over-conservative, such an approach seemed appropriate given the somewhat limited size of our cohort and lack of a replication cohort ascertained according to the same protocol.

## Results

A total of 1,425 subjects from 248 families were recruited to the study (of whom 45.5% were male and 38.6% hypertensive). The median family consisted of 5 subjects. In total, 60% of families comprised between 4 and 6 genotyped and phenotyped members. 71% of families consisted of 2 generations, with remaining families consisting of 3 generations. 16% of families contained assessable sibships only in the proband’s offspring; in the remaining 84% there was an assessable sibship within the proband’s generation. The second phase of cardiovascular phenotyping, requiring electrocardiogram assessment, was completed in 955 family members (449 men and 506 women), 67% of the total cohort. Subjects with structural heart disease on echocardiography (N = 69) and with electrocardiographic abnormalities consistent with previous myocardial infarction (N = 18) were excluded from analysis, delivering a sample of 868 eligible subjects for the genetic analyses. On average, excluded subjects were older, more likely to be hypertensive, diabetic and male. 224 families (395 men and 473 women) were included in the electrocardiographic analyses. The demographics of the population are shown in Table 1.

**Table 1:** Characteristics of the study population (n = 868).

Characteristic	Value
Male, *n* (%)	395 (45.5)
Age in years, mean (±SD)	52.4 (13.5)
Systolic BP, mean in mmHg (±SD)	137 (21)
Hypertension, *n* (%)	349 (42.3)
Antihypertensive treatment, *n* (%)	330 (40.0)
*ECG LVH, *n* (%)	86 (9.9)
Diabetes, *n* (%)	21 (2.4)
Weight, mean in kg (±SD)	76.8 (14.7)
Height, mean in m (±SD)	1.68 (0.1)
Body mass index, mean in kg/m^2^ (±SD)	27.1 (4.8)
Waist-hip-ratio (±SD)	0.87 (0.1)

* ECG left ventricular hypertrophy based on any one of the following criteria: Sokolow-Lyon voltage >35 mm; RaVL >11 mm; Cornell voltage >28 mm in men and >20 mm in women; Cornell product >0.24 mV ms.

The ten SNPs were successfully genotyped in 95.7% ±1.61 participants, with no departures from Hardy-Weinberg equilibrium at the 5% significance level and high concordance of the observed allele frequencies with those reported in the HapMap CEU population (www.hapmap.org) (Table S1). The estimated genotype error rate was <1%. The rs7223247 SNP, in the fourth exon of the *TLCD2* gene, had a minor allele frequency of 0.078 in our total population. The major allele of this SNP is guanine (G) and the minor allele is thymidine (T). Data on ECG, genotype, and all significant covariates included in the final model were present in 708 individuals. The genotype at this SNP was significantly associated with the log-transformed, covariate-adjusted residual Sokolow-Lyon voltage (Table 2). In view of the small number of T/T homozygotes we combined G/T and T/T individuals in the association analyses (that is, a dominant genetic model). The T allele was associated with a higher SLV derived LVM (*P* = 0.0038) (Table 2). Carriers of the T allele had a 0.272 standard deviation higher LVM by SLV. The fitted model implies a 9% difference in the untransformed values between the lowest and highest value genotypes. The genotype of rs7223247 accounted for approximately 1% of the total LVM variance. Following post-test Bonferroni correction (correcting for the 10 genotyped SNPs), rs7223247 remained significantly associated with SLV derived LVM (P = 0.038).

**Table 2:** Association between rs7223247 genotype and Sokolow-Lyon voltage.

	rs7223247 genotype			p-value for GG vs (TG+TT)
	GG	TG	TT	
N	598	99	11	
Sokolow–Lyon voltage	2.24 (0.653)	2.45 (0.581)	2.42 (0.657)	0.0019
Adjusted Sokolow–Lyon voltage	−0.0509 (0.956)	0.214 (0.873)	0.199 (0.775)	0.0038

Figures are mean (standard error).

## Discussion

We have shown that genotype at the rs7223247 SNP in the *TLCD2* gene neighbouring *miR-22* contributes to the variance of left ventricular mass measured by SLV on the ECG. The major G allele at this SNP is associated with lower values of LVM. Carriers of the minor allele had ∼10% higher LVM than non-carriers, and genotype accounted for ∼1% of the population variability in LVM. The rs7223247 SNP is situated in exon 4 of the *TLCD2* gene. *TLCD2* has four exons, and is transcribed on the reverse strand of chromosome 17. The mRNA transcript produced is 625 bp in length, and is entirely derived from a segment of exon 4 that is slightly 3′ (in the direction of transcription) to rs7223247. TLCD2 has been hypothesised to be involved in ceramide synthesis and lipid metabolism, which has been associated with cardiac metabolism and may contribute towards cardiac hypertrophy [26]. rs7223247 is a synonymous SNP, with no obvious involvement in splicing or transcription factor binding. Functional studies will be required to clarify if rs7223247 is associated with quantitative differences in levels of TLCD2 transcription that may in turn be related to variance in LVM, which appears to be the most likely mechanism for the association. An alternative explanation is a *cis-*acting effect of rs7223247 on *miR-22* expression. Although previous studies have demonstrated that SNPs within the pre-miR region can influence miR expression [27], [28], fewer data are available in respect of more distant eQTLs influencing miR expression. Further studies will be required to investigate whether rs7223247 genotype has a *cis*-acting effect altering *miR-22* expression [29].

Multiple hypothesis driven candidate gene studies have investigated LVM previously; however, hypothesis-generating genome-wide association studies (GWAS) are the most comprehensive method of evaluating the genetic effects on LVM. However, results from GWAS focused on LVM have so far been disappointing. A previous echocardiography-based GWAS conducted by Vasan et al. investigated 12,612 individuals, but demonstrated no locus affecting LVM at genome-wide significance [30]. This contradicts recent findings reported by Shah et al. who demonstrated, in a large candidate gene study, 4 loci within 10,256 individuals contributing towards ECG derived LVM [31]. A much smaller Korean GWAS conducted by Hong et al. reported 14 SNPs from 8 genetic loci to be associated with ECG-LVM [32]; however, none of these correlate with the regions detected in the analyses by Shah et al. Our focused investigation of candidate genes within a linked region may be considered complementary to these GWAS approaches. The region of chromosome 17 where *miR-22* is located has not been significantly associated (p<10^−5^) with ECG LVM in these previous studies.

Differences between studies may be explained in part due to the recruitment strategy. Our cohort represents a collection of families recruited via a proband within the upper 5% of the blood pressure distribution and incorporated additional hypertensive individuals in extending nuclear pedigrees. It is thus dissimilar to previous studies, as a third of participants were classified within the upper 5% tail of the blood pressure distribution and overall the prevalence of left ventricular hypertrophy was 30% higher in our cohort compared, for example, with the community-ascertained cohorts studied by Vasan et al. [30]. Moreover, our previous work has shown higher heritability for ECG derived measures of LVM than for echo derived measures; and typically regions showing association with echocardiographic and electrocardiographic LVM have shown little overlap. Taken together, these considerations should increase our power to detect genetic effects related to LVM. However, these hypothesis-generating findings require replication in additional cohorts. Indeed, it would be useful to analyse rs7223247 genotypes within additional cohorts enriched for high blood pressure (or otherwise selected for higher LVM) in future studies.

The conclusions drawn from this study are fully supported by electrocardiographic data, derived from detailed phenotyping methods, accounting for potential confounding variables.The current study does however have certain limitations. As we employed stringent selection criteria, results may not be generalizable for families who are not genetically “loaded” for hypertension. Although significant covariates were adjusted for, anti-hypertensive medications were not specifically adjusted for, which may have a minor confounding effect. It is known that ECG is not the most sensitive method for recording LVM and future studies enrolling hypertensive patients may derive benefit from utilising magnetic resonance imaging to further investigate this association with superior precision.

In summary, we have shown a significant effect of the rs7223247 SNP in the *TLCD2*/*miR-22* region and LVM, in hypertensive families. From a clinical perspective, the magnitude of the association we have described is too small to have any role in genetic risk stratification, for example to guide treatment decisions in patients with borderline hypertension. However, genes in our region of association may be therapeutic targets for preventing cardiac hypertrophy in the context of hypertension.

## Supporting Information

Table S1 SNPs neighbouring miR-22 genotyped in family sample
(DOCX)
