# Supplementary material for: Common Variation Neighbouring Micro-RNA 22 Is Associated with Increased Left Ventricular Mass
Source: PLoS One. 2013 Jan 25;8(1):e55061. doi: 10.1371/journal.pone.0055061 (PMC3555935; doi:10.1371/journal.pone.0055061)
Supplement: Table S1 — SNPs neighbouring miR-22 genotyped in family sample (DOCX) [file pone.0055061.s001.docx]

**Table S1: SNPs neighbouring miR-22 genotyped in family sample**

| **SNP** | **17p13.3 location** | **Number Genotyped (%)** | | **Allele Frequency** | | | | **HW P-value** | | **ECG SNP association**  **P-values** |
| --- | --- | --- | --- | --- | --- | --- | --- | --- | --- | --- |
|  |  | **Total cohort**  **(n=1425)** | **ECG**  **(n=868)** | **Present study** | | **HapMap CEU** | |  |  |  |
|  |  |  |  | **Minor** | **Major** | **Minor** | **Major** | **All** | **Founders** |  |
| rs10852873 | 1551330 | 1362  (95.6%) | 840  (96.8%) | 0.496  (T) | 0.504  (C) | 0.496  (C) | 0.504  (T) | 0.7864 | 0.2661 | 0.029 |
| rs1574525 | 1570425 | 1367  (95.9%) | 843  (97.1%) | 0.298  (G) | 0.702  (A) | 0.323  (G) | 0.677  (A) | 0.9485 | 0.01134 | 0.019 |
| rs4790809 | 1558183 | 1385  (97.2%) | 849  (97.8%) | 0.100  (A) | 0.900  (G) | 0.124  (A) | 0.876  (G) | 0.3682 | 0.4315 | 0.6 |
| rs4790814 | 1568927 | 1376  (96.6%) | 850  (97.9%) | 0.492  (G) | 0.508  (A) | 0.491  (A) | 0.509  (G) | 0.9570 | 0.7174 | 0.62 |
| rs7221974 | 1577334 | 1381  (96.9%) | 846  (97.5%) | 0.266  (G) | 0.734  (A) | 0.204  (G) | 0.796  (A) | 0.8906 | 0.2420 | 0.21 |
| rs7223247 | 1555779 | 1342  (94.2%) | 828  (95.4%) | 0.078  (T) | 0.922  (G) | 0.106  (T) | 0.894  (G) | 0.0204 | 0.1040 | 0.0038 |
| rs7502296 | 1560975 | 1389  (97.5%) | 852  (98.2%) | 0.342  (T) | 0.658  (C) | 0.332  (T) | 0.668  (C) | 0.6336 | 0.1365 | 0.76 |
| rs8076112 | 1558949 | 1377  (96.6%) | 846  (97.5%) | 0.160  (C) | 0.840  (A) | 0.168  (C) | 0.832  (A) | 0.1088 | 0.5239 | 0.35 |
| rs4790812 | 1564885 | 1309  (91.9%) | 814  (93.8%) | 0.311  (A) | 0.689  (G) | 0.241  (A) | 0.759  (G) | 0.0046 | 0.5651 | 0.49 |
| rs9912287 | 1577742 | 1345  (94.4%) | 741  (85.4%) | 0.207  (A) | 0.793  (C) | 0.254  (A) | 0.746  (C) | 0.0249 | 0.3050 | 0.38 |
